# Resilience after adversity: an umbrella review of adversity protective factors and resilience-promoting interventions

**DOI:** 10.3389/fpsyt.2024.1391312

**Published:** 2024-10-04

**Authors:** Biruk Beletew Abate, Ashenafi Kibret Sendekie, Abay Woday Tadesse, Tesfaye Engdaw, Ayelign Mengesha, Alemu Birara Zemariam, Addis Wondmagegn Alamaw, Gebremeskel Abebe, Molla Azmeraw

**Affiliations:** ^1^ College of Medicine and Health Science, Woldia University, Woldia, Ethiopia; ^2^ Department of Clinical Pharmacy, College of Medicine and Health Science, University of Gondar, Gondar, Ethiopia; ^3^ College of Medicine and Health Sciences, Samara University, Samara, Ethiopia; ^4^ Curtin School of Population Health, Curtin University, Perth, WA, Australia

**Keywords:** adversity, resilient, protecting factors, interventions, umbrella review

## Abstract

**Introduction:**

Resilience is the dynamic adaptive process of maintaining or recovering mental health from stressors, such as trauma, challenging life circumstances, critical transitions, or physical illnesses. Resilience after adversity can be fostered through protective factors and the implementation of interventions that promote resilience. Hence, it is essential to investigate both protective and vulnerable factors to reduce the negative effects of unfavorable life events and increase resilience through positive risk-response interventions.

**Objective:**

To assess the effect of previous adversity, protecting factors, and resilience-promoting interventions to possess resilience after adversity in a global context.

**Methods:**

The study included English language articles sourced from PubMed, Embase, Scopus, Web of Sciences, the Cochrane Database of Systematic Reviews, Scopus, and Google Scholar published before 15 April 2024. These articles reported the effect of adversity, protecting factors, and/or resilience-promoting interventions to possess resilience after adversity in a global context without a population age limitation. The quality of the included studies was assessed using the Assessment of Multiple Systematic Reviews. A weighted inverse-variance random-effects model was applied to find the pooled estimates. The subgroup analysis, heterogeneity, publication bias, and sensitivity analysis were also assessed.

**Results:**

A total of 44 articles (n = 556,920 participants) were included in this umbrella review. From the random-effects model analysis, the pooled effect of adversity on the development of resilience was 0.25 (*p* < 0.001). The pooled effects of adversity-protective factors and resilience-promoting interventions after adversity were 0.31 (*p* < 0.001) and 0.42 (*p* < 0.001), respectively. The pooled effects of specific adversity protective factors were 0.26, 0.09, 0.05, 0.34, 0.23, and 0.43 for the availability of support, cognitive ability, community cohesion, positive self-perception, religious involvement, and self-regulation, respectively. The pooled effects of specific resilience-promoting interventions were 0.30, 0.21, 0.51, and 0.52 for cognitive behavior therapy (CBT) interventions, mindfulness-based interventions, mixed interventions, and resilience-promoting interventions, respectively.

**Conclusion:**

The findings of this umbrella review revealed that people who experienced early adversity can develop resilience later in life. The study highlights the need to consider adversity protective factors, such as availability of support (family, friends, and school), cognitive ability, community cohesion, positive self-perception, religious involvement, and self-regulation, and resilience-promoting interventions, including CBT interventions, mindfulness-based interventions, and mixed interventions, to enhance resilience promotion programs.

## Introduction

Adverse childhood experiences (ACEs) are potentially traumatic events that can be categorized as abuse (physical, emotional, and sexual), neglect (physical and emotional), and household challenges (mental illness, parent treated violently, divorce, incarcerated relative, and substance abuse) (1–3). Exposure to ACEs can raise the risk of various health and social problems, including harm, sexually transmitted diseases, maternity and pediatric health issues (teen pregnancy, pregnancy complications, and fetal death), human trafficking, suicide, and chronic diseases such as diabetes, heart disease, and cancer (3).

Researchers and practitioners increasingly recognize ACEs as powerful determinants of health and well-being. Originating from various sources, ACEs can have long-term detrimental effects on physical and mental health. These experiences are associated with an increased risk of developmental delays, learning difficulties, depression, diabetes, heart disease, substance abuse, and other chronic illnesses (3). Victims of Adverse Childhood Experience (ACE) might be subjected to additional harmful stress as a result of past and present traumas, and a graded association was observed between the number of ACE categories and the incidence of adult disorders such as liver disease, cancer, ischemic heart disease, chronic lung disease, and skeletal fractures (4).

The prevalence and impact of ACEs is a global concern, although the extent of the problem varies significantly across different regions and populations. Although data collection methodologies and definitions of ACEs can contribute to inconsistencies in prevalence rates, the available evidence suggests that ACEs are widespread. For instance, studies have reported ACE prevalence rates ranging from 31% to 93.5% in China (5, 6), 46.2% in young Europeans (7), and a substantial 72% to 82% in Sub-Saharan Africa (8).

The primary goal of the investigation into the “Effect of ACEs on Resilience Later in Life” is to clarify the complex connection between early adversity and people’s capacity to overcome, adapt, and thrive. Research suggests that protective factors within the home, school, and community are acknowledged, nurtured, and understood (9–11). Individual resilience is defined as the ability to tolerate, adjust to, and overcome stress and adversity (12). This capacity is fostered by the development of adaptive skills, positive experiences, and supportive relationships (13). Resilience acts as a buffer against the detrimental effects of stress, promoting positive emotional and cognitive development in children (13).

Several factors influence the development of resilience. The duration of adversity, the presence of supportive relationships, and previous experiences with challenges all contribute to a child’s resilience (14). The Centers for Disease Control and Prevention has outlined strategies to mitigate the immediate and long-term consequences of ACEs. These include strengthening economic support for families, promoting violence prevention, ensuring early childhood well-being, enhancing parenting and youth coping skills, fostering supportive relationships, and providing timely interventions (15).

The central objective of resilience researchers is to identify vulnerability and protective factors that influence how individuals respond to adversity. By understanding the psychological, emotional, and social mechanisms underlying resilience, researchers aim to develop targeted interventions. Resilience-promotive or adversity-protective factors include supportive relationships and effective coping strategies (16, 17). Understanding the underlying mechanisms of resilience is crucial for identifying pressing issues such as maternal depression, often linked to factors like family conflict, ineffective coping strategies, and negative parenting practices (18). Although research suggests that adversity-protective factors and resilience-promoting interventions can increase resilience, the findings are inconsistent. This gap emphasizes the need for comprehensive research to inform effective interventions and improve practices for promoting resilience. Therefore, this umbrella review aimed to (I) synthesize the pooled effects of childhood adversity; (II) determine the pooled effect of adversity-protective factors, and (III) examine the pooled effect of resilience-promoting interventions on the development of resilience following childhood adversity.

## Methods

This umbrella review systematically synthesized eligible systematic review and meta-analysis (SRM) reports following established umbrella review methodology (19). Although not pre-registered in PROSPERO, the review protocol has been submitted and is available upon request from the corresponding author. The study adhered to the Preferred Reporting Items for Systematic reviews and Meta-Analyses (PRISMA) guidelines and used excluding criteria based on an earlier study (20).

### Searching strategy and information sources

A comprehensive search was conducted in PubMed, Embase, Web of Sciences, the Cochrane Database of Systematic Reviews, Scopus, and Google Scholar for studies published before 15 April 2024. These studies examined the impact of protective factors and resilience-promoting interventions on resilience development in a global context. No restrictions were placed on the study start year or participant age. Only English language articles were included. The search employed medical subject heading (MeSH) terms, keywords, and their combinations. Additionally, reference lists of retrieved articles were manually searched for potential studies. Five key concepts were identified for each search term, forming the basis of the search strategies.

Concept 1 (adversity): adverse childhood experience, life challenges, unpleasant, misfortune, hardship, distress, suffering, and sorrow. Concept 2 (resilience): resilience, resilien*, recovery, over-coming, resiliency, and adaptive function. Concept 3 (protective factors): religion, spirituality, support, self-esteem, and coping. Concept 4 (interventions): training, CBT, education, mindfulness, counseling, promotion, intervention, and meditation. Concept 5 (SRM): meta-analysis’, ‘systematic review’, and ‘review’.

The literature search was carried out by two reviewers independently, with discrepancies resolved by consensus. Articles with incomplete reported data were handled by contacting corresponding authors. We used the search terms independently and/or in combination using “OR” or “AND” [(“adversity” OR “adverse childhood experience” OR “life challenges” OR “unpleasant” OR “misfortune” OR “hardship” OR “distress” OR “suffering” OR “sorrow”) AND (“resilience” OR “resilien*” OR “recovery” OR “over-coming” OR “resiliency” OR “adaptive function”) AND (“religious” OR “spirituality” OR “support” OR “self-esteem,” OR “coping”) AND (“training” OR”CBT” OR “education” OR “mindfulness” OR “counseling” OR “promotion” OR “intervention” OR “meditate”)]. A sample of the literature search strategy, the PubMed search strategy, developed using a combination of MeSH terms and free texts is presented as a **Supplementary Data Sheet 1** (**Supplementary Table 1**). In addition to the systematic database search, an article search was carried out using the reference list of the included studies and the ‘cited by’ and ‘related articles’ functions of PubMed.

### Study selection/eligibility criteria

Retrieved articles were imported into Endnote v8 for duplicate removal. The screening and selection of studies were conducted in two stages. Two reviewers (BBA and MAB) independently screened titles and abstracts, followed by a full-text assessment using pre-specified inclusion criteria. To be included, SRMs had to report the effects of protective factors and resilience-promoting interventions on resilience development in a global context and be published in English. Additionally, studies were required to (a) present a defined literature search strategy, (b) appraise included studies using a relevant tool, and (c) employ a standardized approach to pooling studies and reporting summary estimates. Studies were excluded for the following reasons: (a) a lack of reporting on the measures of interest, (b) a language other than English, and (c) study type (narrative reviews, editorials, correspondence, abstracts, and methodological studies). Disagreements regarding study inclusion were resolved through consensus among reviewers.

### Quality assessment

The methodological quality of included systematic reviews was assessed independently by two reviewers (AKS and AMK) using the AMSTAR tool (21). AMSTAR evaluates the quality of systematic reviews based on 11 items assessing the rigor of methods used for pooling and summarizing studies. Given its reliability and widespread use, AMSTAR was selected for this review. Studies were categorized as high (8–11), medium (4–7), or low (≤3) quality based on their total AMSTAR score. Only reviews with a medium- or high-quality rating (score ≥4) were included in this study. A predetermined quality threshold was established and agreed upon by all reviewers before the assessment process. Although the quality of the included systematic reviews was assessed, the quality of primary studies within these reviews was not explicitly evaluated for this umbrella review.

### Data extraction

Data from the included SRMs were extracted using a standardized Excel-based data abstraction form. For each SRM, the following information was collected: (a) study identification (first author, publication year), (b) review aim and type, (c) estimate of resilience among adversity-exposed populations, (d) effects of adversity protective factors, (e) effects of resilience-promoting interventions, (f) included primary studies (design type and sample size), (g) sample size of included studies, (h) publication bias assessment methods and results, (i) quality assessment methods and scores, (j) data synthesis method (random or fixed-effects), and (k) authors’ main conclusions (**Table 1**).

**Table 1:** Distribution of the included studies on resilience after adversity: the effect of protecting factors and resilience-promoting interventions, 2024.

SrNo	Author	Year	Design	Number of studies	Sample size	Resilience- promoting interventions	Protective factor	Effect size (95% CI)
**1**	Wan X et al. (31)	2022	SRM	17	4,156	PT growth	Self-regulation	0.448(0.37, 0.519)
**2**	Ang WHD et al. (35)	2021	SRM	22	2,876	Training		0.54(0.28, 0.79)
**3**	Castillo‐González A et al. (45)	2022	SRM	29	2,750			0.03(-0.21, 0.27)
**4**	Han S-J, Yeun Y-R et al. (36)	2023	SRM	15	852	Resilience-promoting interventions		0.59(0.31, 0.86)
**5**	Llistosella M etal. (37)	2023	SRM	16	2,468	CBT interventions		0.58(0.29, 0.87)
**6**	Yule K et al. (30)	2019	SRM	15	8,592		Positive self-perceptions	0.31(0.22, 0.4)
**7**	Yule K et al. (30)	2019	SRM	7	2,178		Positive self-perceptions	0.06(-0.08, 0.21)
**8**	Yule K et al. (30)	2019	SRM	6	1,322		Cognitive ability	0.17(0.01, 0.33)
**9**	Yule K et al. (30)	2019	SRM	6	3,306		Cognitive ability	0.06(-0.02, 0.14)
**10**	Yule K et al. (30)	2019	SRM	12	2,568		Self-regulation	0.45(0.35, 0.53)
**11**	Yule K et al. (30)	2019	SRM	8	4,993		Self-regulation	0.3(0.15, 0.43)
**12**	Yule K et al. (30)	2019	SRM	14	1,881		Coping	0.01(0.23, 9.5)
**13**	Yule K et al. (30)	2019	SRM	6	1,018		Coping	0.03(0.24, 4.33)
**14**	Yule K et al. (30)	2019	SRM	49	69,619		Family support	0.12(0.2,42.63)
**15**	Yule K et al. (30)	2019	SRM	30	26,524		Family support	0.14(0.22, 36.8)
**16**	Yule K et al. (30)	2019	SRM	13	13,494		Parental effectiveness	0.12(0.23, 23.42)
**17**	Yule K et al. (30)	2019	SRM	7	6,216		Parental effectiveness	0.05(0.16,4.33)
**18**	Yule K et al. (30)	2019	SRM	16	50,323		School support	0.13(0.28,25.13)
**19**	Yule K et al. (30)	2019	SRM	5	7,494		School support	0.19(0.24, 2.77)
**20**	Yule K et al. (30)	2019	SRM	15	22,683		Peer support	0.05(0.19, 16.59)
**21**	Yule K et al. (30)	2019	SRM	11	7,916		Peer support	0.04(0.2, 12.76)
**22**	Yule K et al. (30)	2019	SRM	4	4,070		Community Cohesion	0.05(0.43, 2.04)
**23**	Yule K et al. (30)	2019	SRM	6	9,196		Community Cohesion	0.01(0.14, 4.53)
**24**	Yule K et al. (30)	2019	SRM	4	18,587		Extra-curricular activities	0.02(0.06, 3.2)
**25**	Yule K et al. (30)	2019	SRM	2	1,557		Extra-curricular activities	0.03(0.24, 1)
**26**	Yule K et al. (30)	2019	SRM	4	18,544		Religious involvement	0.03(0.06, 2.33)
**27**	Yule K et al. (30)	2019	SRM	2	1,879		Religious involvement	0.01(0.3, 1)
**28**	Liu JJ et al. (38)	2020	SRM	20	-	Resilience-promoting interventions		0.48(0.4, 0.56)
**29**	Lee JH et al. (32)	2013	SRM	5	1,140		Social support	0.41(0.29, 0.53)
**30**	Lee JH et al. (32)	2013	SRM	5	3,916		Positive self-perceptions	0.55(0.44, 0.66)
**31**	Lee JH et al. (32)	2013	SRM	6	2,428		Optimism	0.42(0.34, 0.5)
**32**	Joyce S et al. (39)	2018	SRM	11	839	Resilience-promoting interventions		0.44(0.23, 0.64)
**33**	Joyce S et al. (39)	2018	SRM	11	839	Mixed interventions		0.51(0.12, 0.91)
**34**	Joyce S et al. (39)	2018	SRM	11	839	CBT-interventions		0.27(0.05, 0.5)
**35**	Joyce S et al. (39)	2018	SRM	11	839	Mindfulness-based intervention		0.46(0.1, 0.82)
**36**	Ma L et al. (40)	2020	SRM	38	24,135	CBT-interventions		0.13(0.06, 0.19)
**37**	Lavoie J et al. (33)	2016	SRM	14	12,772		Protective effects	0.47(0.17, 0.7)
**38**	Lavoie J et al. (33)	2016	SRM	14	12,772			0.19(0.05, 0.34)
**39**	Wu Y et al. (41)	2023	SRM	19	2,048	Resilience-promoting interventions		0.3(-0.47, 0.35)
**40**	Schwalm FD et al. (34)	2021	SRM	34	8,721		Religious involvement	0.4(0.32, 0.48)
**41**	Pinto TM et al. (42)	2021	SRM	13	2,799	Resilience-promoting interventions		0.48(0.15, 0.81)
**42**	Liu X et al. (43)	2022	SRM	20	7,988	Mindfulness-based intervention		0(0.5, 1)
**43**	Hodder RK et al. (44)	2017	SRM	13	16,619	Resilience-promoting interventions		0.78(0.6, 0.93)
**44**	Morgan CA et al. (46)	2022	SRM	9	161,164			0(-0.05, 0.5)

### Statistical analysis

Data extracted from the included SRMs using Microsoft Excel were imported into STATA v14.0 for analysis. A combined narrative and quantitative approach was used to summarize review estimates. When multiple estimates were available, a range of estimates and a pooled estimate were presented. Pooled estimates for the effects of adversity, protective factors, and resilience-promoting interventions on later-life resilience were calculated with 95% confidence intervals (CIs) and visualized using forest plots (22). The pooled estimates with 95% CIs were presented using forest plots.

Heterogeneity among the included SRMs was assessed using Galbraith plots, inverse variance (I² statistics), and Cochran’s Q statistic. I² values, ranging from 0 to 100%, quantify the proportion of total variance attributable to heterogeneity (23, 24). I^2^ of 25%, 50%, and 75% thresholds for low, moderate, and high heterogeneity, respectively, with a threshold of I² < 50%, is often considered indicative of low heterogeneity (25, 26). Additionally, τ² was calculated to estimate between-study variance, as I² can be influenced by study size (27). To further explore heterogeneity, 95% prediction intervals (PIs) for the standardized mean difference were calculated, providing a range for potential effect sizes in future studies (27). I², τ², and 95% PIs were computed for each SRM (28).

For heterogeneous data, a DerSimonian–Laird random-effects model was applied. Subgroup analyses were conducted based on protective factors and resilience-promoting intervention types. Sensitivity analysis was performed to evaluate the impact of individual studies on overall estimates. Publication bias was assessed using funnel plots and Egger’s regression test (29).

## Results

**Figure 1:**
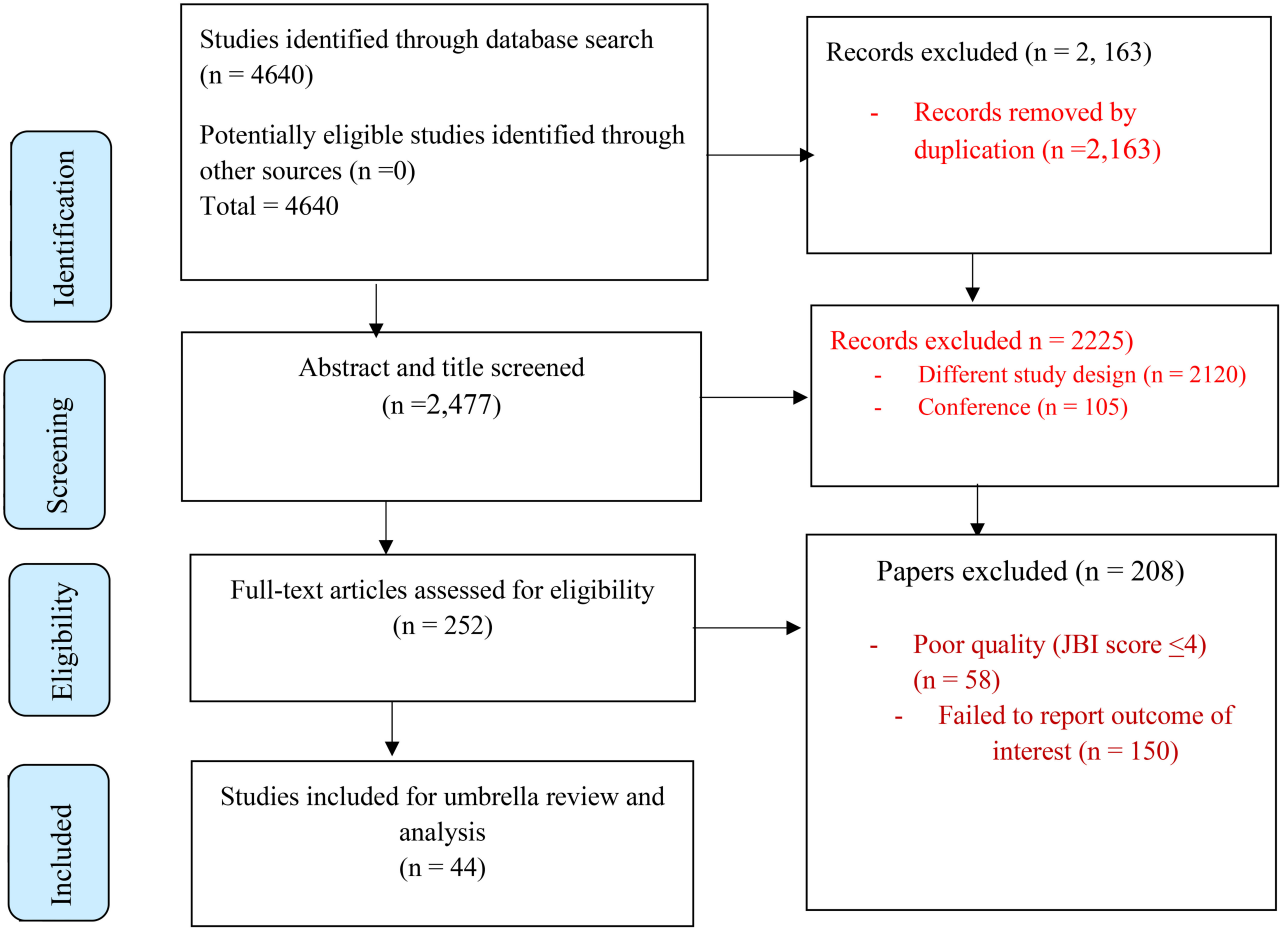
PRISMA-adapted flow diagram showing the results of the search and reasons for exclusion.

A total of 4,640 studies were identified from different databases. After removing duplicates (2,163), 2,477 records remained. Subsequent title and abstract screening resulted in 252 full-text articles being selected for detailed evaluation, 208 of which were subsequently excluded due to poor quality (JBI score <4) (n=58) and a failure to report an outcome of interest (n=150). Finally, 44 articles encompassing 556,920 participants met the inclusion criteria and were included in the final analysis (**Figure 1**).

### Characteristics of the included studies

The number of studies included in each SRM ranged from 2 (30) to 49 (30). The minimum and maximum sample sizes were 1,557 and 69,619, respectively. Out of 44 included SRMs, 27 investigated adversity protective factors for resilience development (Wan X et al. (31), Yule K et al. (30), Lee JH et al. (32), Lavoie J et al. (33) and Schwalm FD et al. (34)). Fourteen SRMs examined the effects of resilience-promoting interventions on resilience development (Wan X et al. (31), Ang WHD et al. (35), Han S-J, Yeun Y-R et al. (36), Llistosella M et al. (37), Liu JJ et al. (38), Joyce S et al. (39), Joyce S et al. (39), Ma L et al. (40), Wu Y et al. (41), Pinto TM et al. (42), Liu X et al. (43), Hodder RK et al. (44)). Five SRMs reported the association between adversity and resilience (Wan X et al. (31), Ang WHD et al. (35), Castillo‐González A et al. (45), Lavoie J et al. (33), Morgan CA et al. (46)) (**Table 1**).

### The effect of protective factors

**Figure 2:**
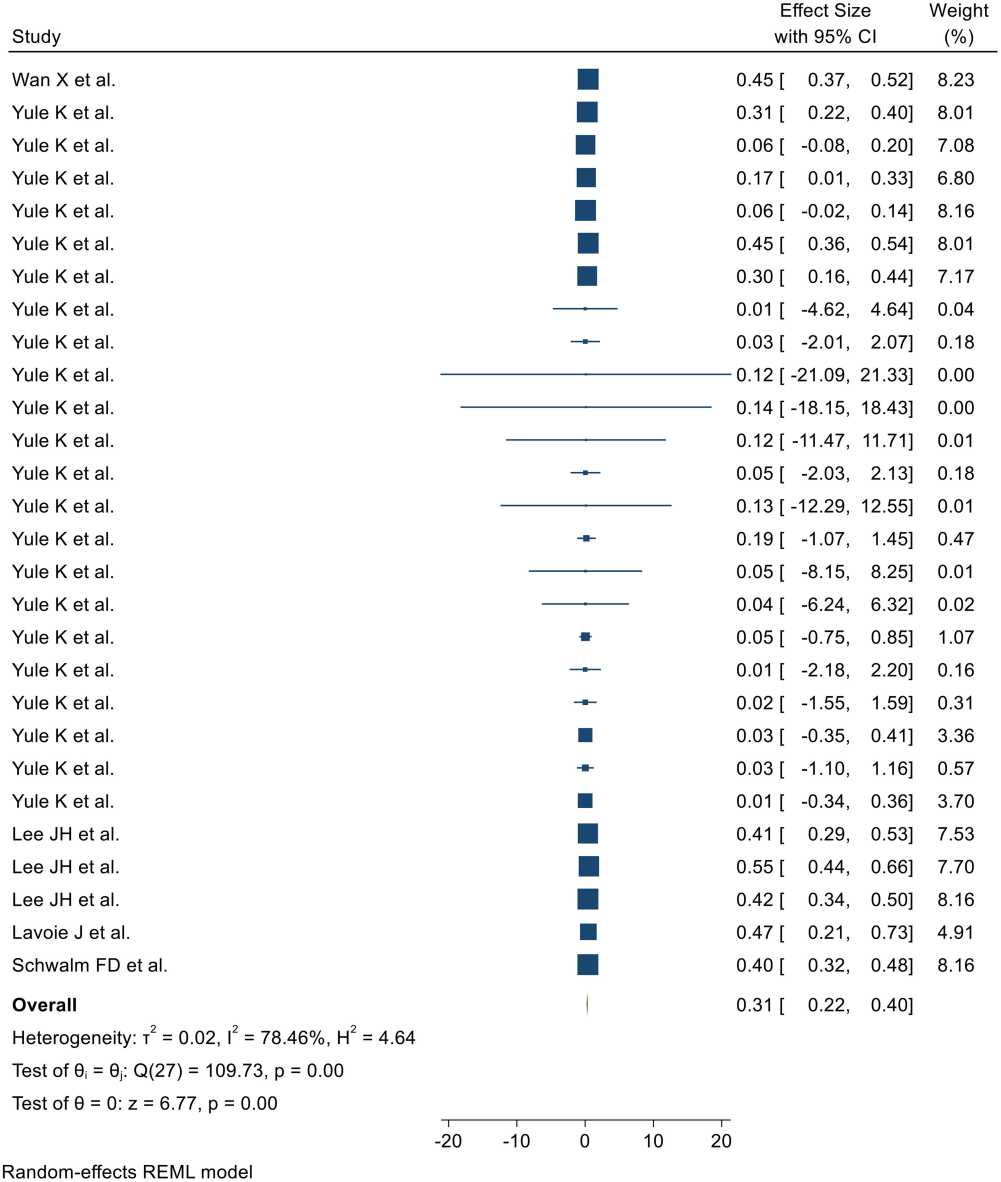
Forest plot showing the pooled effect of these protective factors for the development of resilience after adversity.

The effects of protective factors were determined from 27 SRMs (Wan X et al. (31), Yule K et al. (30), Lee JH et al. (32), Lavoie J et al. (33), and Schwalm FD et al. (34)). The effects of protective factors ranged from 0.01 (95% CI: 0.14, 4.53) (30) to 0.45 (95% CI: 0.37, 0.519) (31). Random-effects model analysis of these studies revealed a pooled effect of protective factors on resilience development following adversity of 0.31 (95% CI: 0.22, 0.40) (I² = 78.46%; *p* < 0.001) (**Figure 2**)

### Heterogeneity outcome

Heterogeneity among the 27 included SRMs was assessed using Galbraith plots and I² statistics. Random-effects meta-analysis revealed a pooled effect of adversity protective factors on resilience development of 0.31 (95% CI: 0.22, 0.40) with substantial heterogeneity (I² = 78.46%, *p* < 0.001), as visualized in the Galbraith plot (**Supplementary Figure 1**).

### Subgroup analysis

Subgroup analysis was conducted based on the protective factor type. Pooled effects for resilience development were 0.26 (95% CI: −0.04, 0.55) for availability of support, 0.09 (95% CI: −0.01, 0.19) for cognitive ability, 0.05 (95% CI: −0.71, 0.80) for community cohesion, 0.34 (95% CI: 0.14, 0.54) for positive self-perception, 0.23 (95% CI: −0.10, 0.57) for religious involvement, and 0.43 (95% CI: 0.37, 0.48) for self-regulation (**Supplementary Figure 2**).

### Publication bias

A funnel plot exhibited a symmetrical distribution (**Supplementary Figure 3**). The Egger’s regression test yielded a value of 0.278, indicating no evidence of publication bias (**Supplementary Figure 4**). Consequently, a trim and fill analysis was not conducted (**Supplementary Figure 5**).

### Sensitivity analysis

A leave-one-out sensitivity analysis was conducted to assess the influence of individual studies on the pooled estimate of protective factors for resilience development. Results indicated that the pooled findings were robust to the exclusion of any single study. The pooled estimate ranged from 0.297 (0.20–0.38) to 0.34 (0.28–0.41) after removing a single study (**Supplementary Figure 6**).

### The effect of resilience-promoting interventions

**Figure 3:**
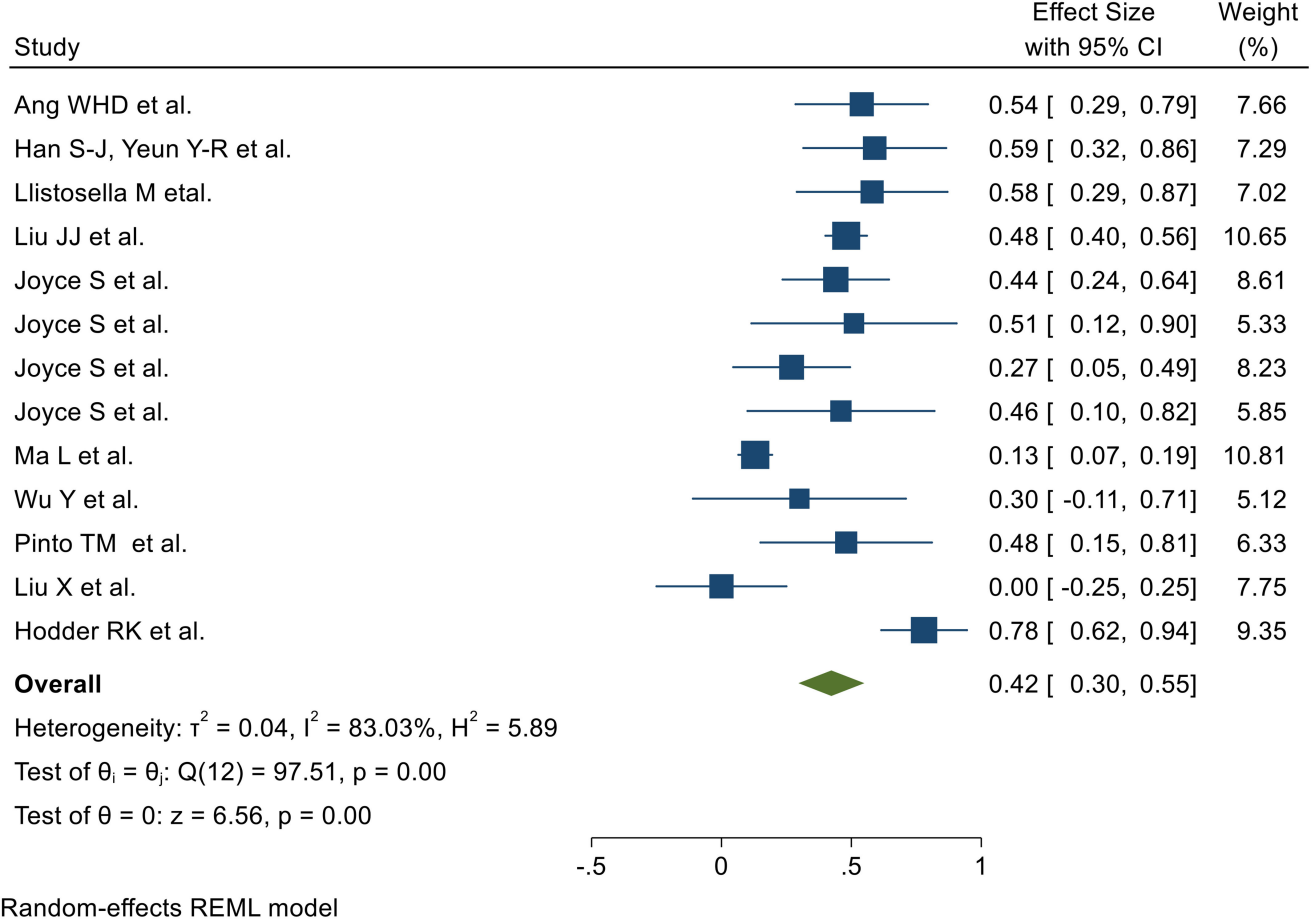
Forest plot showing the pooled effect of resilience-promoting interventions for the development of resilience after adversity.

Fourteen of the 44 included SRMs reported the effects of resilience-promoting interventions on resilience development following adversity (Wan X et al. (31), Ang WHD et al. (35), Han S-J, Yeun Y-R et al. (36), Llistosella M etal. (37), Liu JJ et al. (38), Joyce S et al. (39), Joyce S et al. (39), Ma L et al. (40), Wu Y et al. (41), Pinto TM et al. (42), Liu X et al. (43), Hodder RK et al. (44)). Random-effects model analysis of these studies revealed a pooled effect of resilience-promoting interventions on resilience development of 0.42 (95% CI: 0.30, 0.55), with substantial heterogeneity (I² = 78.46%; *p* < 0.001) (**Figure 3**).

### Heterogeneity outcome

Heterogeneity among the 14 included SRMs was assessed using Galbraith plots and I² statistics (**Supplementary Figure 7**). Random-effects meta-analysis revealed a pooled effect of resilience-promoting interventions on resilience development of 0.42 (95% CI: 0.30, 0.55), with substantial heterogeneity (I² = 78.46%, *p* < 0.001).

### Subgroup analysis

Subgroup analysis was conducted based on the types of resilience-promoting interventions. Pooled effects for resilience development were 0.30 (95% CI: 0.05, 0.55) for CBT interventions, 0.21 (−0.24, 0.66) for mindfulness-based interventions, 0.51 (95% CI: 0.12, 0.90) for mixed interventions, and 0.52 (95% CI: 0.42, 0.62) for resilience-promoting interventions (**Supplementary Figure 8**).

### Publication bias

A funnel plot showed a symmetrical distribution (**Supplementary Figure 9**). The Egger’s regression test value was 0.647, which indicated the absence of publication bias (**Supplementary Figure 10**). Owing to the absence of publication bias, we did not employ a trim and fill analysis (**Supplementary Figure 11**).

### Effects of previous adversity on resilience later in life

**Figure 4:**
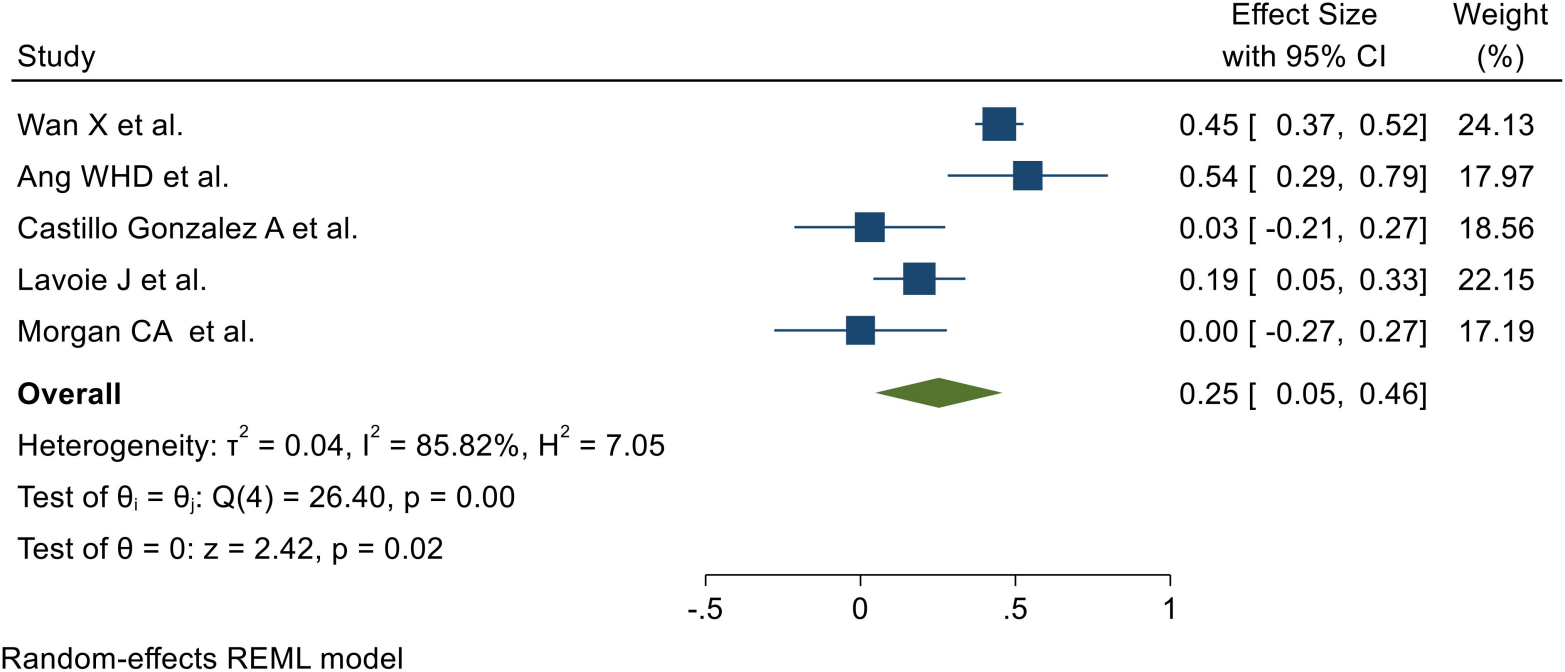
Forest plot showing the pooled effect of adversity for the development of later resilience.

Five SRMs reported the association between previous adversity and later-life resilience (Wan X et al. (31), Ang WHD et al. (35), Castillo‐González A et al. (45), Lavoie J et al. (33), Morgan CA et al. (46)). The random-effects model analysis from those studies revealed that the pooled effect of adversity for the development of resilience after previous adversity was 0.25 (95% CI: 0.05, 0.46) (I^2^ = 85.82%; p < 0.001) (**Figure 4**).

### Heterogeneity outcome

The Galbraith plot and I² were used to assess heterogeneity among the five included SRMs (**Supplementary Figure 12**). Random-effects meta-analysis revealed a pooled effect of adversity on resilience of 0.25 (95% CI: 0.05, 0.46), with substantial heterogeneity (I² = 85.82%; *p* < 0.001). The Galbraith plot also indicated heterogeneity.

### Publication bias

A funnel plot exhibited a symmetrical distribution (**Supplementary Figure 13**). The Egger’s regression test yielded a value of 0.39, indicating no evidence of publication bias (**Supplementary Figure 14**). Consequently, a trim and fill analysis was not conducted.

### Sensitivity analysis

A leave-one-out sensitivity analysis was conducted to assess the influence of individual studies on the pooled estimate of adversity on resilience development. Results indicated that the pooled findings were robust to the exclusion of any single study. The pooled estimate ranged from 0.18 (0.04–0.42) to 0.30 (0.10–0.50) after removing individual studies (**Supplementary Figure 15**).

## Discussion

This umbrella review aimed to assess the effects of adversity, adversity protective factors, and resilience-promoting interventions on resilience development in a global context. Results indicated a significant pooled effect of resilience-promoting interventions on post-adversity resilience. According to Morgan CA et al.’s SRM, a substantial negative correlation exists between ACEs and resilience (46). Children worldwide face various adversities, with some developing resilience and others experience long-term psychological and social challenges. Individuals with ACEs were 63% less likely to exhibit high resilience compared with those without ACEs, emphasizing the importance of identifying factors contributing to resilience development.

The current study found a pooled effect of 0.31 for adversity protective factors on resilience development. These protective factors, including the availability of support, cognitive ability, community cohesion, positive self-perception, religious involvement, and self-regulation, significantly influenced resilience. This finding aligns with the work of Yule K et al. in 2019 (30).

Accordingly, 26% of individuals experiencing childhood hardship developed resilience due to supportive relationships with family, friends, or their school, aligning with Afifi TO and MacMillan HL’s 2011 analysis, “Resilience After Child Abuse: An Examination of Protective Elements” (47). Religious participation also contributed to resilience, with 23% of trauma-exposed individuals reporting increased resilience following religious involvement. These findings support previous studies on the protective effects of parental involvement and religion against behavioral problems in violence-exposed adolescents (48, 49).

To build resilience and mitigate the harmful effects of childhood adversity, future studies should prioritize the development and evaluation of interventions. Universal resilience-focused interventions, particularly CBT, have demonstrated efficacy in reducing ACE-related depressive and anxiety symptoms (50). Early community resilience assets, such as fair treatment, supportive friendships, opportunities for skill development, trusted adult relationships, and positive role models, have been independently linked to positive outcomes (51).

Research indicates that positive early relationships with compassionate adults can mitigate or prevent the harmful effects of ACEs, a form of toxic stress (3). Although ACEs pose significant challenges, some individuals demonstrate resilience by overcoming adversity and achieving personal growth. Emerging research highlights the role of protective factors and resilience in mitigating the negative impacts of ACEs. Resilience theories emphasize individuals’ strengths, including coping strategies (internal) and family and community support (external), in navigating stressful experiences, rather than focusing solely on vulnerabilities (52–54). Positive individual, family, and community characteristics, such as strong family functioning and parental engagement, are linked to better outcomes for children and adolescents exposed to ACEs (55, 56). Strong family functioning can serve as a protective factor against teenage mental health issues, poverty, neighborhood violence, and dysfunctional parental relationships (9–11).

This umbrella review indicates that interventions incorporating mindfulness or CBT techniques can enhance resilience measures. Although our analysis revealed significant pooled effects of resilience-promoting interventions across various subtypes, including CBT, mindfulness-based, and mixed interventions, further research is needed to optimize these approaches.

Previous studies have demonstrated the positive impact of resilience training on psychological well-being (57). Additionally, research suggests that CBT can influence resilience through its impact on life stories (58). A combination of CBT and mindfulness techniques within resilience interventions shows promise, building upon the established efficacy of these approaches in treating mental health conditions such as depression and anxiety (39, 59–61). Moreover, these interventions have been linked to improved overall psychological and physical health (62).

### Implications for research

Protective factors, such as caregiver support, can increase resilience without necessarily directly impacting psychological symptoms, although their relationship to maladjustment warrants further investigation (63). To accurately measure resilience in children exposed to violence, studies should consider both indicators of healthy development and protective factors (63).

Research emphasizes the importance of strong relationships and understanding broader cultural contexts in fostering children’s resilience, calling for improved prevention strategies (64, 65). Effective prevention and health promotion across diverse cultural groups requires understanding how sociocultural factors influence protective factors within families, schools, and peer groups (66). The Resilience Portfolio Model offers a structured framework for examining factors influencing children’s adjustment processes (67, 68).

### Implications for prevention and intervention

This study emphasizes the importance of universal prevention, sensitive caregiving, supportive family relationships, and parental intervention programs in fostering children’s resilience (69). Caregiver programs promoting self-regulation, including emotion socialization strategies, have demonstrated improvements in socio-emotional competencies and reduced behavioral issues among preschoolers (70).

Schools are transitioning from punitive discipline toward promoting healthy development through social emotional learning (SEL) principles, cultivating supportive relationships and individual strengths among students and teachers (71–73). SEL programs, encompassing self-regulation, teacher-student relationships, positive self-perception, and symptom reduction, can be integrated into school-based initiatives (74). Mindfulness-based interventions (75), such as compassion and attention in schools, which teach attention focus and mental/physical self-control (76, 77), have shown benefits for self-perception, well-being, self-regulation, coping, and mental health in children and adolescents (78, 79).

Trauma-sensitive schools adopt a comprehensive approach, including SEL curricula, support services, and targeted interventions, to address the impact of trauma on children’s behavior (80–82). Research affirms the potential of trauma-sensitive schools to enhance children’s functioning, regardless of trauma exposure (82). Although this meta-analysis highlights the significance of protective factors in parenting and school-based programs, their effectiveness in enhancing children’s functioning is often underestimated (83). Program evaluations investigating the impact of self-regulation and supportive teacher-student relationships on children’s health and well-being contribute to resilience research by examining their additive and buffering effects.

The findings of this study should be interpreted with caution due to several limitations. First, the included studies exhibited substantial heterogeneity, indicating variability in the methodologies, populations, and outcomes reported. Although a random-effects model and subgroup analysis were employed to account for this heterogeneity, it is important to acknowledge that the pooled estimate may not accurately represent the true effect size in all contexts. Second, the geographical distribution of the included studies was uneven, with a potential overrepresentation of certain regions. This limitation may restrict the generalizability of the findings to populations from underrepresented geographical areas. Finally, the inclusion of only English language studies might have led to the exclusion of relevant research conducted in other languages. This language bias could potentially limit the comprehensiveness of the evidence base.

## Conclusion and recommendation

This umbrella review demonstrates that individuals exposed to early adversity can develop resilience. Protective factors, including support from family, friends, and school, cognitive abilities, community cohesion, positive self-perception, religious involvement, and self-regulation, are associated with an increased likelihood of developing resilience following adversity. Resilience-promoting interventions, such as CBT and mindfulness-based approaches, can also enhance resilience outcomes. To foster resilience among at-risk youth and their caregivers, concerted efforts to strengthen positive relationships within families, schools, and communities are essential.

## Data Availability

The original contributions presented in the study are included in the article/**Supplementary Material**. Further inquiries can be directed to the corresponding author.
