# Supplementary material for: Resilience after adversity: an umbrella review of adversity protective factors and resilience-promoting interventions
Source: Front Psychiatry. 2024 Oct 4;15:1391312. doi: 10.3389/fpsyt.2024.1391312 (PMC11487322; doi:10.3389/fpsyt.2024.1391312)
Supplement: Supplementary file 1 [file DataSheet1.zip › Supplementary Figures.DOCX]

**Supplementary Files**

Figure S1: Galbraith plot showing heterogeneity the pooled effect of protective factors for the development of resilience after adversity

Figure S2: Subgroup analysis showing heterogeneity the pooled effect of protective factors for the development of resilience after adversity

Figure S3: Funnel plot showing heterogeneity the pooled effect of protective factors for the development of resilience after adversity

**
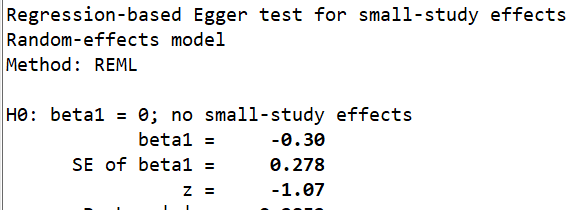
**

Figure S4: Egger test of publication bias the pooled effect of protective factors for the development of resilience after adversity

**
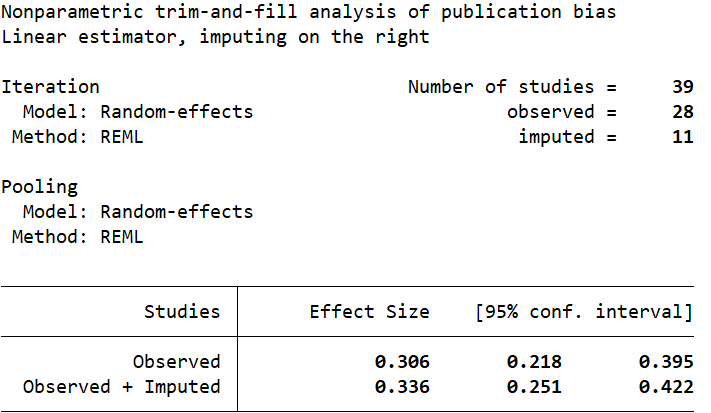
**

Figure S5: Trim and fill analysis the pooled effect of protective factors for the development of resilience after adversity

**Protective factors**


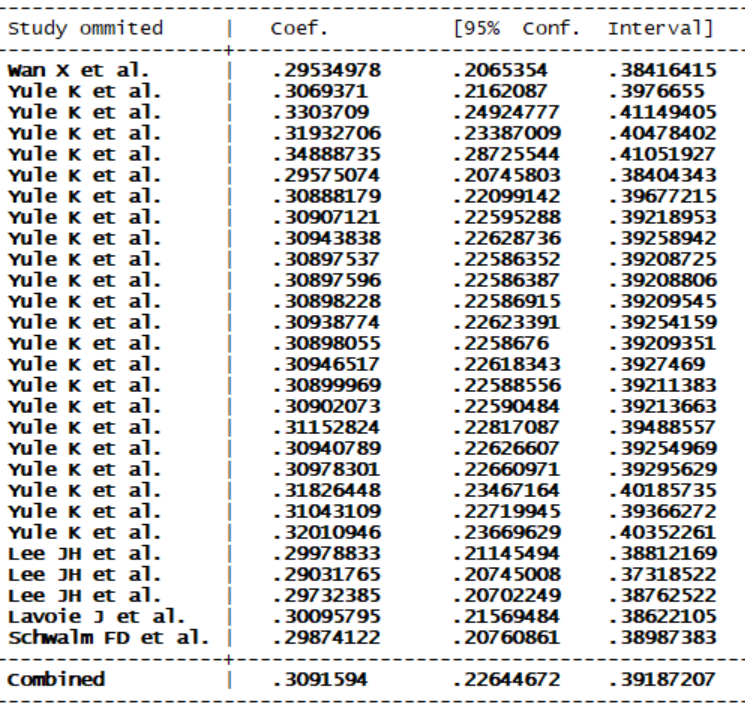


Figure S6: sensitivity analysis the pooled effect of protective factors for the development of resilience after adversity

Figure S7: Galbraith plot for the pooled effect of resilience-promoting for the development of resilience after adversity

Figure S8: Subgroup analysis for the pooled effect of resilience-promoting for the development of resilience after adversity

Figure S9: Funnel plot showing publication bias for the pooled effect of resilience-promoting for the development of resilience after adversity

**
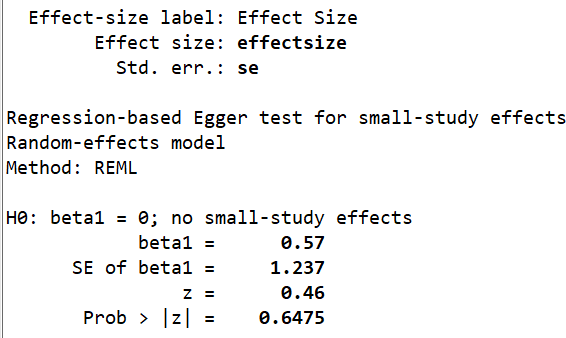
**

Figure S10: Egger test showing publication bias for the pooled effect of resilience-promoting for the development of resilience after adversity

**
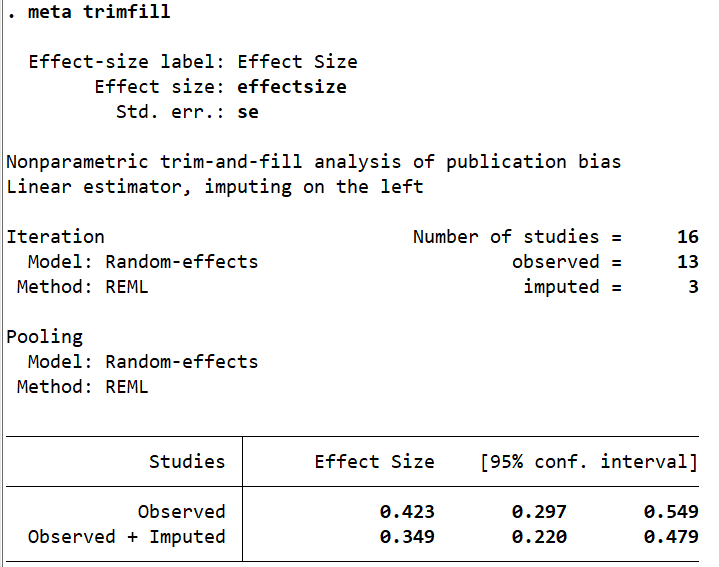
**

Figure S11: Trim-and-fill analysis for the pooled effect of resilience-promoting for the development of resilience after adversity

Figure S12: Galbraith plot for the pooled effect of adversity for the development of resilience after adversity

Figure S13: Funnel plot for the pooled effect of adversity for the development of resilience after adversity

**
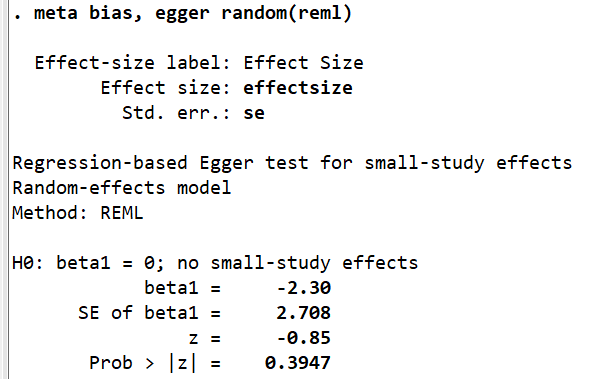
**

Figure S14: Funnel plot for the pooled effect of adversity for the development of resilience after adversity

**Sensitivity analysis**

**
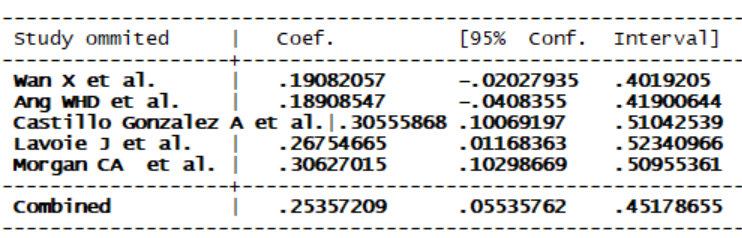
**

Figure S15: Sensitivity analysis for the pooled effect of adversity for the development of resilience after adversity
