# Supplementary material for: Resilience after adversity: an umbrella review of adversity protective factors and resilience-promoting interventions
Source: Front Psychiatry. 2024 Oct 4;15:1391312. doi: 10.3389/fpsyt.2024.1391312 (PMC11487322; doi:10.3389/fpsyt.2024.1391312)
Supplement: Supplementary file 1 [file DataSheet1.zip › Supplementary Table.DOCX]

Supplementary Table 1:

| Concept 1 | Concept 2 | Concept 3 | Concept 4 | Concept 5 |
| --- | --- | --- | --- | --- |
| Adversity | Resilience | protective factors | Interventions | Systematic Review OR |
| Adverse childhood experience | resilien* | Religious | Training, | Meta analysis OR |
| life challenges, | Recovery | Spirituality | CBT | Meta-analysis |
| Unpleasant experience, | Over-coming | Support | Education | Review |
| Misfortune, | Resiliency | Self-esteem | Mindfulness |  |
| Hardship, | Adaptive function | Coping | Counselling |  |
| Suffering, |  |  | Promotion |  |
| Sorrow |  |  | Meditate |  |

PubMed search engine: ((“adversity” OR “adverse childhood experience” OR “life challenges” OR “unpleasant” OR “misfortune” OR “hardship” OR “distress” OR “suffering” OR “sorrow” AND (“resilience” OR “resilien*” OR “recovery” OR “over-coming” OR “resiliency” OR “adaptive function”) AND (“religious” OR “spirituality” OR “support” OR “self-esteem,” OR “coping”) AND (“training” OR”CBT” OR “education” OR “mindfulness” OR “counseling” OR “promotion” OR “intervention” OR “meditate”)).
